# Decidual NR2F2-Expressing CD4^+^ T Cells Promote TH2 Transcriptional Program During Early Pregnancy

**DOI:** 10.3389/fimmu.2021.670777

**Published:** 2021-05-18

**Authors:** Yikong Lin, Di Zhang, Yangyang Li, Yunyun Li, Bin Li, Meirong Du

**Affiliations:** ^1^ Laboratory for Reproductive Immunology, NHC Key Lab of Reproduction Regulation (Shanghai Institute of Planned Parenthood Research), Shanghai Key Laboratory of Female Reproductive Endocrine Related Diseases, Hospital of Obstetrics and Gynecology, Fudan University Shanghai Medical College, Shanghai, China; ^2^ Department of Obstetrics and Gynecology, Shanghai Ninth People’s Hospital, Shanghai Jiao Tong University School of Medicine, Shanghai, China; ^3^ Department of Immunology and Microbiology, Shanghai Institute of Immunology, Shanghai Jiao Tong University School of Medicine, Shanghai, China; ^4^ Department of Obstetrics and Gynecology, Guangzhou First People’s Hospital, School of Medicine, South China University of Technology, Guangzhou, China

**Keywords:** CD4^+^T cell, NR2F2, GATA-3, pregnancy, maternal-fetal immune tolerance

## Abstract

A unique immunotolerant microenvironment with Th2 bias in the decidua provides an essential security for successful pregnancy. The disorganized maternal-fetal immune tolerance contributes to more than 50% of unexplained recurrent spontaneous abortion (RSA). How the Th2 bias is developed at the maternal-fetal interface remains undefined. NR2F2, a member of steroid/thyroid nuclear receptor superfamily, is endowed with diverse importance in cell-fate specification, organogenesis, angiogenesis, and metabolism. Here, we showed that NR2F2 was absolutely highly expressed in decidual CD4^+^T(dCD4^+^T) cells, but not in peripheral circulating CD4^+^T cells during early pregnancy. Decidual NR2F2-expressing CD4^+^T cells dominantly produced Th2 cytokines. In unexplained RSA patients, NR2F2 expression in dCD4^+^T cells was significantly decreased, accompanied with disordered phenotype of dCD4^+^T cells. Furthermore, overexpression of NR2F2 promoted the Th2 differentiation of naive CD4^+^T cells. Immunoprecipitation experiment confirmed the binding relationship between GATA-3 and NR2F2, which implied GATA-3 may be an important interactive element involved in the immunoregulatory process of NR2F2. This study is the first to reveal a previously unappreciated role for NR2F2-mediated dCD4^+^T cells in maternal-fetal immune tolerance and maintenance of normal pregnancy, in the hope of providing a potential biomarker for prediction and prevention of clinical unexplained RSA.

## Introduction

The fantastic phenomenon is worthy to be explored that semi-allogeneic fetus can avoid the attack or rejection from the maternal immune system during successful pregnancy (1). Complicated immunoregulation is required to accurately create an immune-tolerant microenvironment at the fetal-maternal interface and maintain the process of pregnancy (2, 3). Once the maternal-fetal immune tolerance is disrupted, various pregnancy-related complications may be elicited, such as recurrent spontaneous abortion (RSA), pre-eclampsia, and fetal intrauterine growth restriction (4, 5). Among them, spontaneous abortion is the most common complication of gestation, occurring in about 15% of human pregnancies. Accumulating evidences have proved CD4^+^T cells are crucial in inducing and maintaining maternal-fetal tolerance. Some cohort studies demonstrate spontaneous abortion is correlated with the decreased proportions of decidual regulatory CD4^+^T cells (Tregs). Moreover, increased decidual CD4^+^T help (Th) 1/Th2 ratio and elevated Th17 proportions are detected in RSA. The excessive infiltration of effector CD4^+^T cells can elicit adverse pregnancy outcome (6–8). All the published data and hypothesis imply a unique characteristic in decidual resident CD4^+^ T cells (9, 10). Whether a representative immune marker or transcriptional regulatory molecule exists in decidual CD4^+^T cells (dCD4^+^ T) to explain the specific decidual phenotype remains unknown.

NR2F2 (nuclear receptor subfamily2, group F genes), also known as COUP-TF2 (chicken ovalbumin upstream promoter-transcription-factor), is extensively characterized as a transcription factor and belongs to the large steroid/thyroid nuclear receptor superfamily. The ligand for NR2F2 has not been identified. NR2F2 is endowed with diverse physiological importance in cell-fate specification, organogenesis, angiogenesis, and metabolism, as well as a variety of diseases (11, 12). The high expression of NR2F2 in developing spinal motor neurons indicates its regulatory roles in neuron differentiation. It is also expressed in the developing eye, developing olfactory bulb and forebrain (13). NR2F2 is reported to be closely related to tumor metastasis and metastasis regulation (14). In female reproductive system, NR2F2 is a key transcription factor for trophoblast syncytization and is necessary to maintain normal reproductive function of women. Placenta malformation and embryo implantation dysfunction were detected in NR2F2 deficient mice (15). While the expression and function of NR2F2 in decidual immune cells has not been reported yet.

In the present study, we first detected the expression of NR2F2 in dCD4^+^ T cells and peripheral CD4^+^ T (pCD4^+^ T) cells. Interestingly, significant difference was observed between them. Then, the number of NR2F2^+^ dCD4^+^ T cells in normal pregnancies (NP) and RSA were compared. Next, we analyzed the cytokine profile of NR2F2^+^ and NR2F2^-^ dCD4^+^ T cells. Moreover, we investigated the role of NR2F2 in promoting the Th2 differentiation, accompanied with the inhibition of Th1 bias *via* the overexpression of NR2F2 in naive CD4^+^ T cells. Finally, we observed the relationship between GATA-3 and NR2F2. Our data provide evidence that the transcriptional factor NR2F2 plays an important role in the maintenance of successful pregnancy through the regulation of dCD4^+^ T cells differentiations and functions, which provides a new insight to explain how the unique characteristics of dCD4^+^T cells are formed.

## Materials and Methods

### Reagents and Human Samples

Human decidual tissue samples were obtained from healthy women (terminated for nonmedical reasons, had at least one successful pregnancy and no history of spontaneous abortions, gestational age 6–12weeks, n = 30) undergoing elective termination of pregnancy. Decidual tissue samples were also obtained from women with unexplained RSAs that occurred during the first trimester of pregnancy (The diagnostic criteria of RSA utilized in this study is patients undergoing spontaneous abortion who also had a history of two or more consecutive spontaneous abortions before 12 weeks gestation without known causes, and excluding those resulting from endocrine, anatomic, genetic abnormalities, infection, etc. gestational age 6–12 weeks, n= 20). The peripheral blood leukocytes were collected at the same time. The demographic and obstetrical characteristics of enrolled participants in NP and RSA groups are summarized in **Table 1**. In addition, umbilical cord blood was collected from women with full-term pregnancy (gestational age 40-42 weeks, n = 6). All procedures were approved by the Human Research Ethics Committee of the Obstetrics and Gynecology Hospital of Fudan University (No. Kyy2016-4) (Shanghai, China). All participants provided written prior informed consent. All methods were conducted in accordance with the approved guidelines.

**Table 1:** Demographic and obstetrical characteristics of enrolled participants.

Subjects	NP	RSA	*p*
Number	30	20	ns
Age mean(years)^a^	28.37±0.57	29.20±0.50	ns
Age range(years)	21-32	22-35	–
Previous spontaneous abortion (number)^a^	–	1.70±0.21	–
Pregnancy week (samples were collected)^a^	7.07±0.17	7.12±0.22	ns
Treatment history	–	–	–

a Median ± standard error of the mean (SEM); ns, not significant.

### Isolation of Mononuclear Cells From the Decidua, Peripheral and Umbilical Blood

Decidual lymphocytes and peripheral blood leukocytes were isolated as previously described (16, 17). Briefly, the endometrial tissues of the first trimester were minced (2–3-mm pieces) and digested with 1.0 mg/ml collagenase type IV (0.1%; Sigma-Aldrich, USA) and 300 μg/ml DNase I (Sigma–Aldrich, USA) for 30-60 min at 37°C. The dispersed cells were then filtered through 100-, 300- and 400-mesh wire sieves. Cells were re-suspended in phosphate-buffered saline (PBS), layered on a discontinuous Percoll density gradient (20%/40%/60%; GE Healthcare, U.S.A.) and centrifuged for 20 min at 800×*g*. Lymphocytes were isolated from the 40%/60% Percoll interface, and both were washed twice in PBS. Peripheral blood mononuclear cells (PBMC) and umbilical cord  blood mononuclear cells (UBMC) were isolated using Ficoll (GE Healthcare, U.S.A.) density gradient centrifugation (20 min, 800×*g*).

### Sorting of Total CD4^+^T Cells and Naive CD4^+^T Cells

The isolated mononuclear lymphocytes from both the decidua and peripheral blood were directly stained for sorting. For FACS sorting, they were incubated with conjugated mouse anti-human antibodies, including CD3-FITC (BioLegend, UK) and CD4-APC (BioLegend, UK), for 30 min at 4°C. After incubation, they were treated with LIVE/DEAD^®^ Fixable Aqua Dead Cell Stain (Invitrogen Life Technologies, U.S.A.) for 10 min. CD3^+^CD4^+^ cells were sorted on a BD FACS Aria-II machine to obtain a purity > 95%. The other mononuclear lymphocytes from the decidua and PBMC were sorted with CD4 MicroBeads (MiltenyiBiotec, Germany), according to the manufacturer’s instructions. To collect the naive CD4^+^T cells, the mononuclear lymphocytes from umbilical blood were sorted with human naive CD4 MicroBeads (MiltenyiBiotec, Germany), according to the manufacturer’s instructions.

### mRNA-Seq Data Analysis

For transcriptomic data, statistical analysis was performed in the R version 4.0.3. Differential expression was computed with limma, and the moderated t-test was used for each comparison. False discovery rate (FDR)-adjusted p-values were calculated with the Benjamini–Hochberg method. We considered transcripts as differentially expressed if the adjusted p-value was <0.05 and the Log_2_(Fold Change) >1. Volcano plot and heatmap with hierarchical clustering was performed using ggplot2 and pheatmap package. Differentially expressed genes between different groups were subjected to functional enrichment analyses and GO analyses to illustrate their functional characteristics and mechanism respectively, using clusterProfiler and pathview package with cut-off values of adjusted p-value <0.05.

### Th1 and Th2 Cell Differentiation

CD4^+^ naive T cells isolated from UBMC were stimulated with anti-CD3 plus anti- CD28 mAbs (R&D, U.S.A.) at a cell to bead ratio of 1:1. For Th1 differentiation, IL-12(10 ng/ml, Peprotech, U.S.A.) and anti-IL-4 (10μg/ml, Biolegend, UK) was added to the culture system. Cells were collected after 5 days stimulation. For Th2 differentiation, CD4^+^ naive T cells were treated with IL-4(20 ng/ml, Peprotech, U.S.A.) and anti-IFN-γ (10μg/ml, Biolegend, UK) and cells were collected 1 week later. The collected cells were evaluated by flow cytometry analysis of intracellular cytokines after polyclonal stimulation.

### Flow Cytometry

Cell surface and intracellular molecular expressions were evaluated by flow cytometry using CytoFLEX (Beckman Coulter, U.S.A.). Fluorescein-conjugated mouse anti-human antibodies were used, including CD3-FITC/PE, CD4-FITC/APC, IFN-γ-PE-CY7/PE, TNF-α-PE-CY7, IL-4-PE/PE-CY7/BV421, IL-5-BV421, IL-13-BV421, T-bet-PE-CY7 and GATA-3-BV421 (Biolegend, UK). Anti-human antibodies NR2F2(Abcam, UK) were binding with fluorescent agent APC (Biolegend, UK) according to the manufacturer’s introduction. For cell-surface staining, single-cell suspensions were stained on ice for 30 min in PBS with 1% fetal bovine serum (FBS). For intracellular staining, cells were fixed and permeabilized using the Fix/Perm kit (Biolegend, UK). To detect intracellular cytokines, CD4^+^T cells were stimulated for 4 h with phorbol 12-myristate 13-acetate (PMA; 1 μg/mL; Sigma) and ionomycin (1 μg/mL; Sigma), and 4 h with GolgiStop (1 μL/mL; BD Biosciences) in a round-bottom 96-well plate. Thereafter, cells were harvested, stained for surface expression, and then fixed and permeabilized for intracellular staining. Flow cytometry data was analyzed using FlowJo software (BD, UK) and CytoExpert software (Beckman Coulter, U.S.A.).

### Plasmid Overexpression and Lentivirus Infection

NR2F2 and GATA-3 overexpressed plasmid and negative control plasmid (Ctrl) were transfected into 293T cells by liposome transient transfection. Transfected cells were incubated for 24 h at 37°C and then collected for further study. Naive CD4^+^T cells were infected with NR2F2 lentivirus specialized for suspension cells (NM_138554, Shanghai Genechem Co., Ltd.), according to the manufacturer’s instructions.

### Exogenous and Endogenous Co-Immunoprecipitation

For exogenous co-immunoprecipitation, 293T cells were washed with 1X PBS and PBS was clearly removed. Then, 300μl lysis buffer was added. After lysis, centrifugation was performed at 12000rpm/10min at 4°C, and supernatant was taken as input and the others were considered as samples. 1μg anti-HA or anti-Flag antibody (Cell Signaling Technology, U.S.A.) was added to samples and incubated overnight at 4°C. 10 μl protein A/G-plus agarose beads (Promega, U.S.A) were added to each tube sample respectively and rotated for 1h at 4°c. After 6 times wash with wash buffer, loading buffer was added for western detection. For endogenous co-immunoprecipitation, dCD4^+^ T cells were cultured to 5-10 million and washed once with 1X PBS. 100μl RIPA was added to lyse the cells for about 1 hour. Protease inhibitors (Sigma, U.S.A.) were added to the lysate. After lysis, centrifugation was performed at 12000rpm/10min. Supernatant was taken as input and the others were divided into two parts. One part was added with 1μg anti-GATA-3 (Santa Cruzes, U.S.A), and the other part was added with 1μg IgG. Samples were incubated overnight at 4°C. Then washed protein A/G beads (Promega, U.S.A) were added into the samples (5-10μl beads per sample) for 1-2h at 4°C. After incubation, RIPA was used to wash off the non-specific binding protein. Finally, the the remaining RIPA was removed and loading buffer was added for further use.

### Western Blotting Analysis

After denaturation, equal amounts of protein were separated *via* SDS-polyacrylamide gel electrophoresis (PAGE) before wet transfer onto polyvinylidene difluoride membranes. Nonspecific binding sites were blocked by incubating the membranes with 5% bovine serum albumin in Tris-buffered saline with 0.1% Tween 20 (TBS-T) for 1 h. Then, the membranes were incubated overnight at 4°C with primary antibodies (1:1000) against HA (1:1000, Cell Signaling Technology, U.S.A.), Flag (1:1000, Cell Signaling Technology, U.S.A.) and NR2F2 (1:1000, Abcam, U.S.A.). Subsequently, membranes were incubated with appropriate horseradish peroxidase-conjugated anti-rabbit (1:5000, Arigo, China) or anti-mouse IgG secondary antibodies (1:5000, Arigo, China) for 1 h at room temperature. After three washes with TBS-T, immunopositive bands on the blots were visualized by using the enhanced chemiluminescence detection system (Amersham Imager600, GE, USA).

### Statistical Analysis

Prism 6 software (GraphPad) was used for data analysis. Statistical significance was determined using Student’s *t*-test for 2-group or one-way ANOVA for multiple group comparisons. All the data were proved to be normal distributed according to the Kolmogorov-Smirnov test. The data were presented as mean ± SEM. Statistical significance was attained when P < 0.05.

## Results

### Transcriptional Factor Related-Gene Expression Profile Between Decidual CD4^+^T Cells and Peripheral CD4^+^T Cells From NP or RSA

To assess the signatures of gene expression in dCD4^+^ T cells of the first trimester, we performed high-throughput mRNA-Seq for paired dCD4^+^ T cells and pCD4^+^ T cells from the same participant of NP. At the same time, dCD4^+^ T cells derived from RSA and NP were compared *via* mRNA-seq.

**Figure 1:**
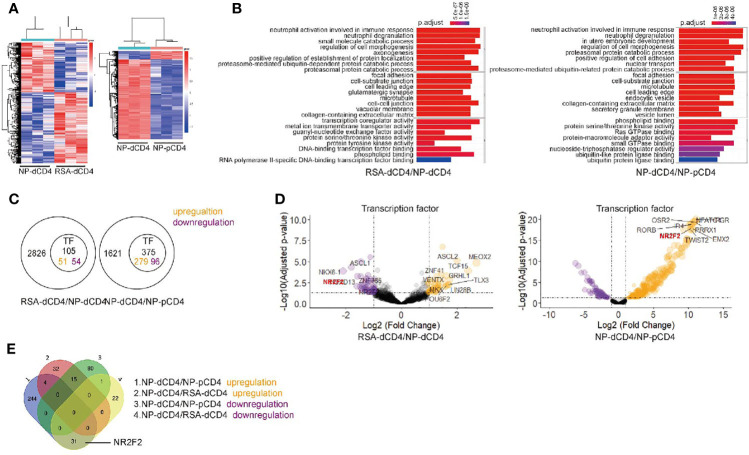
Transcriptional analysis of CD4^+^ T cells from peripheral blood and decidua. **(A)** Heatmap result of an unsupervised hierarchical clustering of genes that is significantly different (*p *< 0.01) in dCD4^+^ T cell samples from RSA compared with dCD4^+^ T cells from NP (left). Heatmap result of differential gene in samples of dCD4^+^ T cells compared with samples of pCD4^+^ T cells from NP (right). Each column represents a patient (blue: NP-dCD4^+^ T cells, red: RSA- dCD4^+^ T or NP-pCD4^+^ T cells), and each row represents a gene. The heatmap indicates the level of row normalized gene expression. Red = high expression; Blue = low expression. **(B)** GO analysis of differentially expressed genes. **(C)** 51 upregulated and 54 downregulated transcriptional factor genes in 2826 differentially expressed genes of early RSA dCD4^+^ T cells. And 279 upregulated and 96 downregulated transcriptional factor genes in 1621 differentially expressed genes of NP dCD4^+^ T cells. **(D)** Volcano plot and **(E)** Venn diagram of differentially expressed transcriptional factor genes; orange and purple points mark the genes with significantly increased or decreased expression, respectively, in samples of dCD4^+^ T cells from RSA compared with dCD4^+^ T cells from NP (FDR < 0.01, left). Volcano plot in samples of dCD4^+^ T cells compared with samples of pCD4^+^ T cells from NP (FDR < 0.01, right). The x-axis shows Log_2_ (Fold Change) in expression, and the y-axis shows the Log_10_ (Adjusted p-value) of a gene being expressed differentially. In both data sets, NR2F2 is the top-ranked gene.

We first demonstrated the differentially expressed genes between dCD4^+^ T cells from RSA and NP by means of a heatmap (left). The differential genes between dCD4^+^ T cells and pCD4^+^ T cells from NP were also depicted in the right heatmap (**Figure 1A**). We observed a total of 2826 differentially expressed genes, with 1666 genes being upregulated and 1160 genes being downregulated in dCD4^+^ T cells from RSA compared with NP. 1621 differentially expressed genes were discovered with 1482 genes being upregulated and 139 genes being downregulated in dCD4^+^ T cells compared with pCD4^+^ T cells. GO analysis illustrated that differentially expressed genes were mainly involved in neutrophil activation and immune response of Biological Process (**Figure 1B**). Then we filtered the transcriptional factors in these differential genes. As shown in **Figure 1C**, 51 upregulated and 54 downregulated transcriptional factor genes were found in early RSA dCD4^+^ T cells compared with NP. Moreover, 279 upregulated and 96 downregulated transcriptional factor genes were found in NP- dCD4^+^ T cells compared with pCD4^+^ T cells from the same participant. With the combination result of Volcano plot (**Figure 1D**) and Venn diagram (**Figure 1E**) of differentially expressed transcriptional factor genes, we found NR2F2 was the most prominent gene which showed higher abundance in dCD4^+^T compared with pCD4^+^T cells and were downregulated in abortion dCD4^+^ T cells compared with NP. Accordingly, NR2F2 was selected as a candidate molecule related to immunoregulation.

### The Differential Expression of NR2F2 Between Decidual CD4^+^T Cells and Peripheral CD4^+^T Cells From NP or RSA

**Figure 2:**
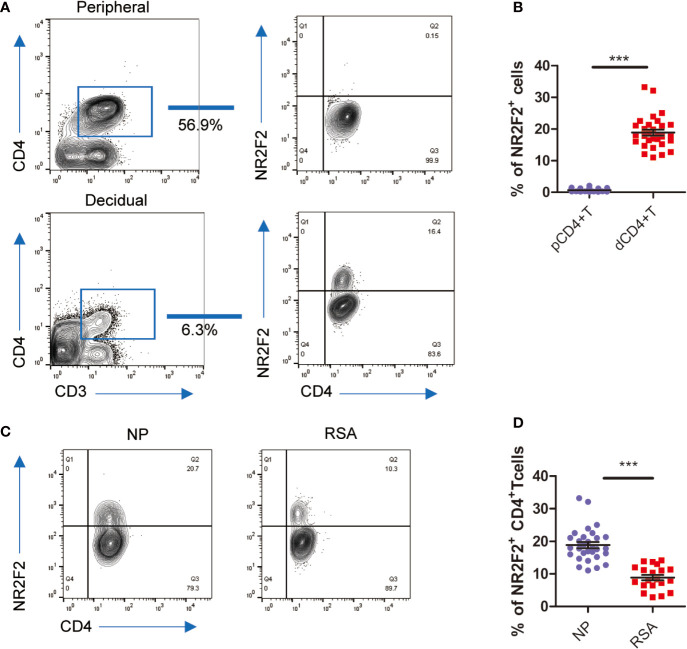
NR2F2 expression in decidual CD4^+^ T cells during early pregnancy. **(A)** Representative image showing the analysis of NR2F2 expression in dCD4^+^ T and pCD4^+^ T cells during the first trimester. **(B)** Relative number of NR2F2^+^ CD4^+^ T cells in gated CD3^+^CD4^+^decidual and peripheral immune T cells (n=30). Data are presented as the mean ± SEM. ***P < 0.001. **(C)** Representative image showing the analysis of NR2F2 expression in dCD4^+^ T cells from NP and RSA during the first trimester. **(D)** Relative number of NR2F2^+^ CD4^+^ T cells from NP(n=30) and RSA (n=20). Data are presented as the mean ± SEM. ***P < 0.001.

To confirm the data from bioinformatics analysis, we first analyzed the expression of NR2F2 in dCD4^+^ T cells. The results in **Figure 2A** showed that approximately 15% of dCD4^+^ T cells expressed NR2F2. We also detected the expression of NR2F2 on pCD4^+^T and found only 0.1% positive percentage (**Figure 2A**). NR2F2^+^ CD4^+^ T cells increased almost 100-fold changes in decidua over peripheral blood, suggesting a possibility that NR2F2 may play a role in forming a decidual specific phenotype of CD4^+^T cells (**Figure 2B**). To obtain further evidence for the role of NR2F2 in dCD4^+^ T cells function during early pregnancy, NR2F2 expression was detected in dCD4^+^ T cells derived from NP and RSA. Compared with NP, the percentage of NR2F2^+^ dCD4^+^ T cells was significantly decreased in RSA (**Figures 2C, D**), suggesting the essential roles of NR2F2^+^ dCD4^+^ T cells in successful pregnancy.

### Decidual NR2F2^+^ CD4^+^T Cells Display the Features of Th2 Phenotypes

**Figure 3:**
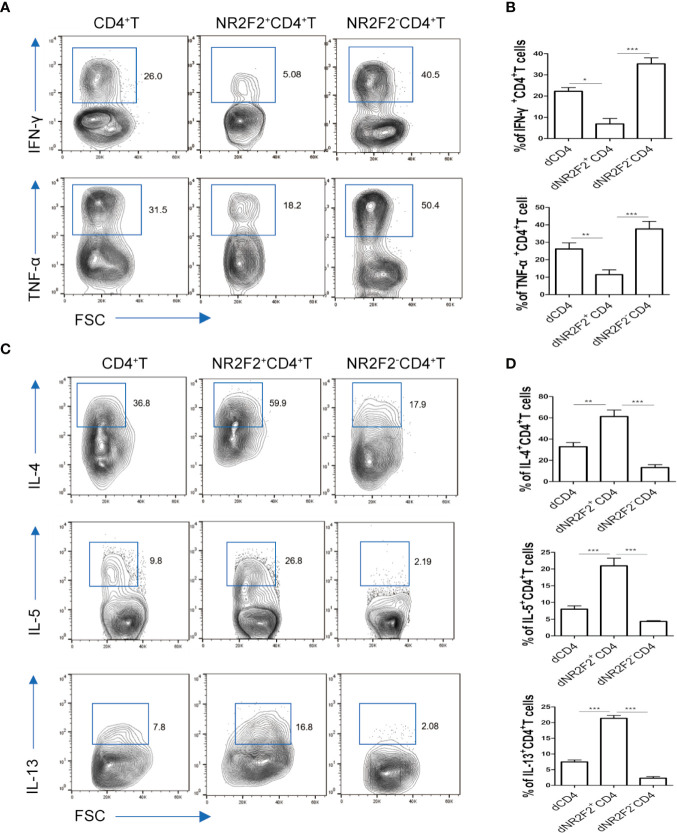
NR2F2^+^ decidual CD4^+^ T cells display a Th2 shift. The relative amounts of Th1-type cytokines **(A, B)** and Th2-type cytokines **(C, D)** in gated total dCD4^+^ T cells, NR2F2^-^dCD4^+^ T cells and NR2F2^+^ dCD4^+^ T cells, respectively, are shown. Data in the right are presented as the mean ± SEM(n=10). *P < 0.05, **P < 0.01, and ***P < 0.001.

To explore whether there is a correlation between NR2F2 expression and the functional status of dCD4^+^ T cells, the secretion of immune-related cytokines was assessed by FACS. As shown in **Figure 3A**, the production of Th1-type TNF-α and IFN-γ was obviously lower in NR2F2^+^ dCD4^+^ T cells compared with the corresponding NR2F2^-^ dCD4^+^ T cell subpopulation (**Figures 3A, B**). On the contrary, the abundance of Th2-type cytokines IL-4, IL-5 and IL-13 was notably high in NR2F2^+^ dCD4^+^ T cells (**Figures 3C, D**). Thus, dCD4^+^ T cells expressing NR2F2 are more advantageous to Th2 bias at the maternal-fetal interface than NR2F2^-^ dCD4^+^ T cells.

### NR2F2 Promotes Th2 Cell Differentiation and Attenuates Th1 Bias

**Figure 4:**
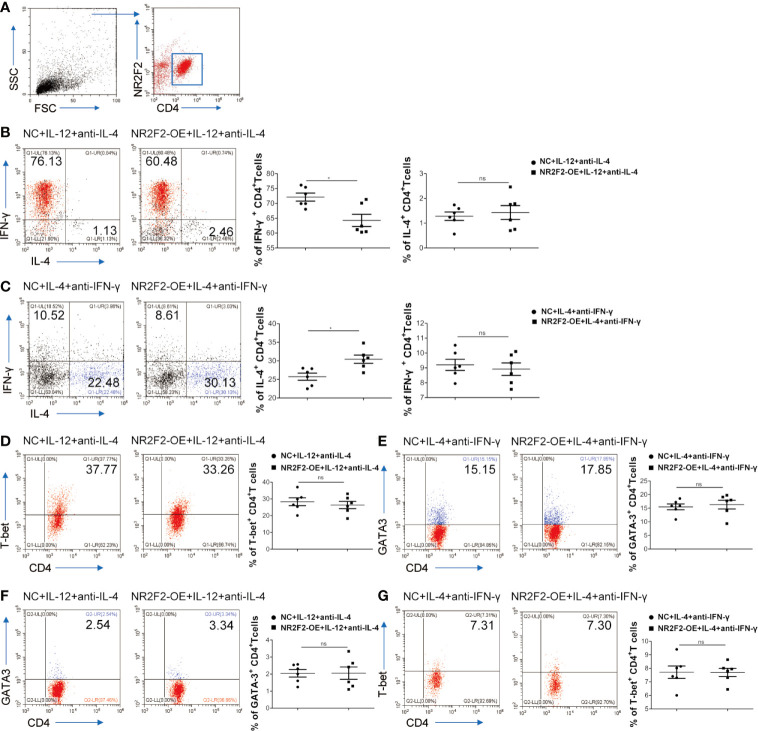
NR2F2 promotes Th2 differentiation with the inhibition of Th1 skewing. Naive CD4^+^ T cells from UBMC were infected with NR2F2 overexpressed-lentivirus which also expressed GFP. **(A)** Gating strategy of naive CD4^+^ T cells from UBMC, CD4^+^ GFP^+^ cells were used for further analysis. **(B)** UBMC naive CD4^+^ T cells were *in vitro* cultured in the presence of anti-CD3 plus anti- CD28 mAbs and of IL-12 and anti-IL-4 for Th1 differentiation. After 5 days, cells were harvested and evaluated by flow cytometry. **(C)** UBMC naive CD4^+^ T cells were *in vitro* treated with IL-4 and anti-IFN-γ for Th2 differentiation and cells were collected 7 days later for flow cytometry detection. **(D)** The expression of T-bet was detected during Th1 differentiation by flow cytometry. **(E)** The expression of GATA-3 was detected during Th2 differentiation by flow cytometry. **(F)** The expression of GATA-3 was detected during Th1 differentiation by flow cytometry. **(G)** The expression of T-bet was detected during Th2 differentiation by flow cytometry. Data are presented as the mean ± SEM (n = 6). *P < 0.05; ns, not significant.

To assess a direct role of NR2F2 in CD4^+^T cell differentiation, NR2F2 was overexpressed in naive CD4^+^T cells isolated from UBMC *via* viral infection which also expressed GFP. Gating strategies were shown in **Figure 4A**. Naive CD4^+^T cells were cultured in the presence of IL-12 and anti-IL-4 to mimic the process of Th1 differentiation *in vitro*. FACS analysis demonstrated that, compared to the CD4^+^T cells infected with control lentivirus, there was a significantly lower frequency of CD4^+^IL-4^-^IFN-γ^+^ Th1 cells in the NR2F2 overexpressed groups (**Figure 4B**). While no obvious differences of IL-4 expression were detected between these two groups, probably due to the dim production during Th1 differentiation (**Figure 4B**). We further examined whether NR2F2 can promote the Th2 bias. Naive CD4^+^T cells were stimulated with IL-4 and anti-IFN-γ to simulate the Th2 differentiation together with NR2F2 overexpression or not. As expected, the differentiation of CD4^+^IL-4^+^IFN-γ^-^ Th2 cells was dramatically increased with the upregulation of NR2F2 (**Figure 4C**). The production of IFN-γ during Th2 differentiation was also calculated, while there was no statistical significance (**Figure 4C**). It is worth noting that there was no significant difference in the expression of T-bet or GATA-3 between the NR2F2-overexpressed CD4^+^T cells and control group whether in the process of Th1 or Th2 differentiation (**Figures 4D, E**). Moreover, the proportions of GATA-3 in Th1 skewing and T-bet in Th2 bias showed no visible variance between the two groups (**Figures 4F, G**). These findings demonstrate that NR2F2 can help to promote naive CD4^+^T cells differentiation to Th2 without altering the expression level of classic Th differentiation related transcription factors, such as T-bet and GATA-3.

### NR2F2 Binds to GATA-3 in Decidual CD4^+^ T Cells

**Figure 5:**
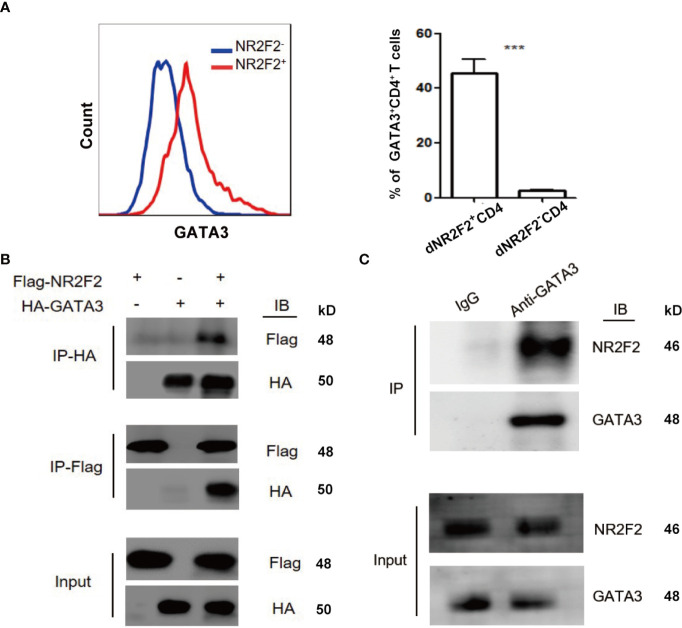
NR2F2 is associated with GATA-3 in decidual CD4^+^ T cells. **(A)** Representative and quantitative images of GATA-3 proportions in NR2F2^+^ and NR2F2^-^ **(B)** Representative images of exogenous immune co-precipitation between NR2F2 and GATA-3 in 293T cells which infected with plasmid of NR2F2-Flag or GATA-3-HA for 24h. **(C)** Representative images of endogenous immune co-precipitation between NR2F2 and GATA-3 in dCD4^+^ T cells. ***P < 0.001.

GATA-3 is widely accepted as a master transcription factor for Th2 skewing. As mentioned above, NR2F2 failed to alter the expression of GATA-3 during Th2 differentiation. We speculated that NR2F2 could promote the transcriptional activity of GATA-3 *via* direct interaction. We therefore investigated the relationship between NR2F2 and GATA-3. The result in **Figure 5A** implied higher enrichment of NR2F2 in GATA-3^+^CD4^+^ T cells compared with GATA-3 negative subset. Exogenous and endogenous co-immunoprecipitation were uesd to further confirm the interaction between these two transcriptional factors. As shown in **Figure 5B**, we detected the exogenous interaction of GATA-3 and NR2F2 in 293T cells 24h after the transfection with GATA-3 and NR2F2 plasmids. We also performed endogenous co-immunoprecipitation in dCD4^+^T cells and the result revealed the binding association between them (**Figure 5C**). These data suggest that NR2F2 may promote the activity of GATA-3 *via* the direct interaction to involve into the regulation of Th2 bias of dCD4^+^ T cells.

## Discussion

CD4^+^ T cells are thought to play a pivotal role in the establishment and maintenance of pregnancy (9). Some subsets of T cells are thought to protect the placenta from immune rejection and facilitate embryo implantation, while others are considered to be the main culprits for some pathological pregnancies (1). The disturbed subset balance and cytokine secretion profile are closely associated with adverse pregnant outcome (18). Effector CD4^+^T cells are divided into multiple subsets characterized by the presence of specific transcription factor and cytokine production, mainly including Th1, Th2, Th17, and Treg cells. Their differentiation is controlled by the following lineage-specific master transcription factors: T-bet for Th1, GATA-3 for Th2, ROR-γt for Th17, and Foxp3 for Treg (19).

In 1990s, researchers firstly suggested that successful pregnancy in mice was associated with a predominant Th2 cytokine profile and that Th1 cytokines were detrimental to pregnancy (20, 21). The dysregulation expression of the Th1-type cytokine TNF-α was shown to lead to the fetal loss in mice (22). With the deepening of research, compelling evidence from human clinical trials reveals the prominent Th2 type response at fetal-maternal interface may contribute to pregnancy maintenance, development of the placenta, and survival of the fetus (23). Moreover, a decreased production of Th2 cytokines and a marked Th1 bias by decidual T cells of women with RSA was observed (24, 25). Fetus, carrying father’s antigen, is a classic semi-allograft. The Th1-type cytokines, which promote allograft rejection, may compromise pregnancy, whereas the Th2-type cytokines, by inhibiting Th1 responses, seems to be central for the induction and the maintenance of allograft tolerance and therefore may improve fetal survival (26). Leukemia inhibitory factor (LIF) is essential for embryo implantation. Th2 cytokine environment can protect trophoblast functions *via* the upregulation of LIF (27). However, the committed Th1 polarization blocking Tregs differentiation can trigger antigen specific fetal loss in RSA patients (28, 29).

Skewing CD4^+^T cell toward a Th2 phenotype provides an alternative, less potentially embryotoxic differentiation state for CD4^+^T cells in comparison to Th1 cells, and seems to be crucial in maternal immune adaption, yet underlying mechanisms remain a great extent obscure (30, 31). A range of recent studies focused on the mechanism of decidual Th2 dominance. For example, hormones could be responsible for the cytokine profile of the T cells, because progesterone is a potent inducer of Th2 cytokines of decidual T cells (32). The inhibitory checkpoint proteins TIM-3, PD-1and CTLA-4 are also reported to play crucial roles in Th2 dominance during early pregnancy (9, 33). However, none of them can explain the Th2 bias from the upstream signaling pathway. As dCD4^+^ T cells are highly differentiated and demonstrate a unique transcriptional profile characterized by various transcriptional factors, a specific upstream regulatory marker of dCD4^+^ T cells remains to be explored.

To discover the regulatory marker, high-throughput mRNA-Seq was performed for dCD4^+^ T cells derived from RSA and NP. Another was utilized between paired dCD4^+^ T cells and pCD4^+^ T cells from the same participant of NP. 2826 and 1621 differential genes were separately screened out from these two bioinformatic results. Among them, there were 105 and 375 transcription factors with differences, respectively. After intersection, 31 differential TFs (including NR2F2) were left which showed downregulation in abortion dCD4^+^ T cells compared with NP and upregulation in normal dCD4^+^ T compared with normal pCD4^+^ T cells. Thinking of the higher expression of NR2F2 in dCD4^+^T compared with pCD4^+^ T cells and the decreased expression of NR2F2 in abortion dCD4^+^ T cells compared with NP, we focused on NR2F2 for further study.

NR2F2 is expressed in the mesenchymal tissue of many organs that require mesenchymal-epithelial interactions for their development, suggesting a very important role during organogenesis (13, 34). NR2F2 is also observed in tumor infiltrating tissue and participates in metastasis regulation (14). At the fetal-maternal interface, NR2F2 is highly expressed in decidual stromal cells and is a key transcription factor for trophoblast syncytization (35). Placenta malformation and embryo implantation dysfunction are detected in NR2F2 deficient mice (15). Published literatures demonstrate the critical function of NR2F2 during the sexual differentiation of female embryo (36, 37). However, the expression and function of NR2F2 in dCD4^+^ T cells has not been reported before. In this study, we demonstrated that approximately 15% of dCD4^+^ T cells expressed NR2F2. While the expression in pCD4^+^ T cells was rare. NR2F2 positive subset displayed high levels of Th2-type cytokine production but low levels of Th1-type cytokine production. Interestingly, dCD4^+^ T cells from RSA showed a downregulation of NR2F2. Our data demonstrated that NR2F2 may be conducive to a Th2-predominant environment and used as an important marker for dCD4^+^ T cells. Here, we found that NR2F2 overexpression promoted Th2 differentiation and potentiated the inhibition of Th1 bias. It is generally accepted that GATA-3 is a master transcription factor for Th2 skewing. We speculated that NR2F2 could mediate Th differentiation *via* the interaction with GATA-3. Results from exogenous and endogenous co-immunoprecipitation confirmed the interaction between these two transcriptional factors.

**Figure 6:**
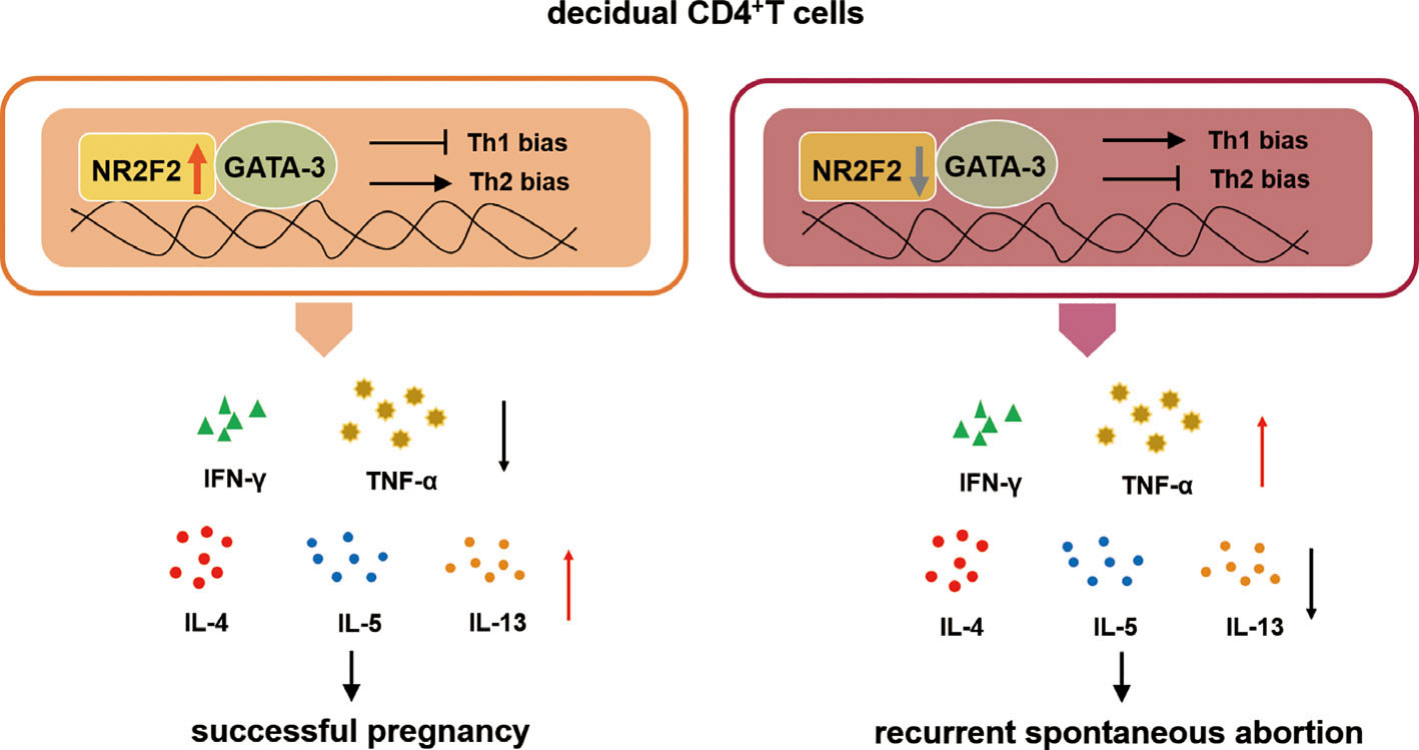
The role of NR2F2 in mediating decidual Th2 bias and pregnancy maintenance. The transcription factor NR2F2 is dominantly expressed in decidual CD4^+^T cells during early pregnancy. The upregulated expression of NR2F2 promotes Th2 differentiation with the inhibition of Th1 skewing *via* binding GATA-3. The population of decidual NR2F2^+^CD4^+^T cells displays Th2-like phenotype, producing higher level of Th2 cytokines with lower level of Th1 cytokines. In RSA patients, the expression of NR2F2 in decidual CD4^+^T cells is decreased, failing to induce Th2 differentiation *via* binding GATA3.

Collectively, our study demonstrates that NR2F2 is preferential expressed in dCD4^+^ T cells compared with peripheral blood during early pregnancy. The upregulated expression of NR2F2 promotes Th2 differentiation with the inhibition of Th1 skewing *via* binding GATA-3. The population of decidual NR2F2^+^CD4^+^T cells displays Th2-like phenotype, producing higher level of Th2 cytokines while lower level of Th1 cytokines. In RSA patients, the expression of NR2F2 in decidual CD4^+^T cells is decreased, failing to induce Th2 differentiation *via* binding GATA-3. These data indicate that deficient expression of NR2F2 in dCD4^+^T cells may be the potential cause of pregnancy failure. The binding relationship between NR2F2 and GATA-3 may help to explain the unique Th2 dominance in dCD4^+^ T cells (**Figure 6**).

## Data Availability Statement

The datasets presented in this study can be found in online repositories. The names of the repository/repositories and accession number(s) can be found in the article.

## Ethics Statement

The studies involving human participants were reviewed and approved by the Human Research Ethics Committee of the Obstetrics and Gynecology Hospital of Fudan University (No. Kyy2016-4). The patients/participants provided their written informed consent to participate in this study.

## Author Contributions

YKL and DZ designed and performed experiments and drafted the manuscript. YaL and YuL analyzed and interpreted the data. MD was responsible for the conception and design of this project. MD and BL revised the manuscript and provided overall direction. All authors contributed to the article and approved the submitted version.

## Funding

The work is supported by the National Key R&D Program of China (2017YFC1001403), the National Nature Science Foundation of China (31970859 and 81630036), International cooperation project between Macao and Shanghai Municipal Commission of science and technology (20410760300), The Strategic Collaborative Research Program of the Ferring Institute of Reproductive Medicine Supported by, Ferring Pharmaceuticals and Chinese Academy of Sciences (FIRMX200504).

## Conflict of Interest

The authors declare that the research was conducted in the absence of any commercial or financial relationships that could be construed as a potential conflict of interest.
